# On multi-marker tests for association in case-control studies

**DOI:** 10.3389/fgene.2013.00252

**Published:** 2013-12-16

**Authors:** Margaret A. Taub, Holger R. Schwender, Samuel G. Younkin, Thomas A. Louis, Ingo Ruczinski

**Affiliations:** ^1^Department of Biostatistics, Johns Hopkins UniversityBaltimore, MD, USA; ^2^Mathematical Institute, Heinrich Heine University DüsseldorfDüsseldorf, Germany

**Keywords:** genome-wide association studies, linkage disequilibrium, multi-marker tests, multiplicity adjustment, single nucleotide polymorphisms

## Abstract

Genome-wide association studies (GWAs) have identified thousands of DNA loci associated with a variety of traits. Statistical inference is almost always based on single marker hypothesis tests of association and the respective *p*-values with Bonferroni correction. Since commercially available genomic arrays interrogate hundreds of thousands or even millions of loci simultaneously, many causal yet undetected loci are believed to exist because the conditional power to achieve a genome-wide significance level can be low, in particular for markers with small effect sizes and low minor allele frequencies and in studies with modest sample size. However, the correlation between neighboring markers in the human genome due to linkage disequilibrium (LD) resulting in correlated marker test statistics can be incorporated into multi-marker hypothesis tests, thereby increasing power to detect association. Herein, we establish a theoretical benchmark by quantifying the maximum power achievable for multi-marker tests of association in case-control studies, achievable only when the causal marker is known. Using that genotype correlations within an LD block translate into an asymptotically multivariate normal distribution for score test statistics, we develop a set of weights for the markers that maximize the non-centrality parameter, and assess the relative loss of power for other approaches. We find that the method of Conneely and Boehnke (2007) based on the maximum absolute test statistic observed in an LD block is a practical and powerful method in a variety of settings. We also explore the effect on the power that prior biological or functional knowledge used to narrow down the locus of the causal marker can have, and conclude that this prior knowledge has to be very strong and specific for the power to approach the maximum achievable level, or even beat the power observed for methods such as the one proposed by Conneely and Boehnke (2007).

## Introduction

Genome-wide association studies (GWAs) are a prominent approach to search for single-nucleotide polymorphisms (SNPs) associated with disease or other phenotypes. To date, results from more than 1000 GWAs have been reported, identifying over ten thousand DNA loci to be statistically associated with one or more of hundreds of phenotypes investigated (http://www.genome.gov/gwastudies). Typically test statistics and *p*-values are reported for each marker on the genomic array, and genome-wide significance for a SNP is declared if the *p*-value after Bonferroni correction is below a threshold for a desired family-wise error rate. Commercially available genomic arrays interrogate the genotypes of individuals at hundreds of thousands or even millions of loci, and *p*-values less than 5×10^−8^ are usually required to achieve genome-wide significance. Obviously, these levels of significance are difficult to reach unless the signal is very strong or the sample size is very large. However, the correlation between neighboring markers in the human genome due to linkage disequilibrium (LD) can be incorporated into statistical tests, and thereby increase the power to detect association under the same family-wise error rate.

Reducing test-multiplicity by taking advantage of the observed marker correlation in LD blocks has been a very active field of research. Haplotype-based methods can be an attractive option to decrease the testing burden (Schaid et al., 2002; Chapman et al., 2003), especially in settings where genetic diversity between subjects is low and/or markers are densely typed. However, most approaches avoid the phasing step for haplotype estimation and use the observed genotypes and/or the respective marginal test statistics and *p*-values instead to generate a single test statistic and *p*-value for the entire LD block. The approaches most similar to the traditionally employed Bonferroni method are those that estimate the “effective number of tests” based on the correlation structure and use those instead of the actual number of tests to control for the family-wise error rate (Nyholt, 2004; Li and Ji, 2005; Moskvina and Schmidt, 2008). Fisher's inverse chi-square test statistic (Fisher, 1932) is another choice to quantify departure from randomness in a set of multiple *p*-values. However, for correlated data such as the *p*-values stemming from the markers in an LD block the inference has to be based on a proper null distribution generated either by permutations (Chapman and Whittaker, 2008) or adjustments to the degrees of freedom in the χ^2^distribution (Makambi, 2003; Chai et al., 2009). Other methods based on the observed genotypes include some traditional multivariate procedures such as Hotelling's *T*^2^-test (Xiong et al., 2002), principal components analysis (Horne and Camp, 2004; Gauderman et al., 2007) and Fourier transformations (Wang and Elston, 2007), but also concepts borrowed from the statistical learning and regularization literature, such as kernel methods (Schaid et al., 2005; Kwee et al., 2008; Mukhopadhyay et al., 2010; Wu et al., 2010; Pan, 2011), penalized regression (Basu et al., 2011), and the LASSO (Shi et al., 2007; Wu et al., 2009). Further, biostatistical concepts employed include latent variables (Wang et al., 2009a), empirical Bayes methods (Goeman et al., 2006), likelihood ratio tests that simultaneously compare genotype means and variances across cases and controls (Wang et al., 2009b), and even hybrids that combine several of those approaches (Pan et al., 2010). While the above methods are based on the observed data only, other approaches also include additional information such as publicly available data bases (Li et al., 2009) or gene sets and ontologies (Wang et al., 2007; Chasman, 2008; Holden et al., 2008; O'Dushlaine et al., 2009). The results of some comparisons of multi-marker tests in case-control studies to detect association with SNP sets have been reported, for example by Chapman and Whittaker (2008) and Ballard et al. (2010).

Despite the advances made in methods development for multi-marker tests and the additional power that can be gained, the standard approach to analyze GWAs data still is to carry out single marker tests with Bonferroni correction. The somewhat limited use of the novel statistical methods is arguably due in part to the fact that some of these methods can be computationally demanding or that open source software is not always available. However, there are powerful multi-marker tests that are very easy to implement and scale, including the approaches proposed by Seaman and Müller-Myhsok (2005) and Conneely and Boehnke (2007). Both methods are based on marginal score tests for each SNP, and the authors demonstrate how genotype correlations within an LD block translate into an asymptotically multivariate normal distribution for the test statistics, with a variance-covariance derived from the estimates of LD. As an alternative to computationally intensive permutation tests, Seaman and Müller-Myhsok (2005) propose to sample from this multivariate distribution to calculate the statistical significance of an observed test statistic, while Conneely and Boehnke (2007) propose to directly use the multivariate cumulative distribution function to calculate *p*-values, particularly for the multi-marker test based on the maximum of the absolute values of the observed marginal test statistics. Computational procedures to assess multivariate normal cumulative distribution functions are readily available, for example as implemented in the statistical software environment R (Genz and Bretz, 2009; Genz et al., 2011). Both of these approaches are completely data driven and do not require prior biological knowledge or external reference data.

In what follows, we quantify the maximum power achievable for multi-marker tests to detect association in case-control studies, which relies on the hypothetical assumption that the locus of the causal marker in an LD block is known. Similar to the derivations in Seaman and Müller-Myhsok (2005) and Conneely and Boehnke (2007) we show that genotype correlations within an LD block translate into an asymptotically multivariate normal distribution for the score test statistics, and develop a set of weights for the markers that maximize the non-centrality parameter in the overall test statistic. We assess the relative loss of power of some alternative, data driven, and thus practical methods without such prior knowledge. We also use some simulations to explore the effect on the power that prior knowledge used to narrow down the locus of the causal marker has, and how much of the maximum achievable power it can reach.

## Methods

### Score test statistics and correlation structures

In a case-control setting we assume the retrospective risk relation to be

(1) $$\pi_{x}=\text{Pr}(G=1|x)=F\text{​}\left(\mu_{G}+\theta_{G}x\right),$$

where *x* ∈ {0, 1} is the fixed binary disease status indicator, and *G* is a function of the genotype that specifies the genetic model. In the following we assume that *G* ∈ {0, 1}, for example encoding a dominant model for a bi-allelic marker (the more general coding is considered in the supplementary materials), but for simplicity still refer to *G* as the genotype. In this setting *G* is the random variable with E(*G*) = π, the relative frequency of *G* = 1 in the study population. As usual, the parameters μ_*G*_ and θ*_G_* describe the relationship between *x* and π via the link *F*, with θ*_G_* being the parameter of interest. We denote the disease status indicator for individual *i* ∈ {1, …, *n*} by *x_i_*, and the genotype for individual *i* by *g_i_*. Thus, ∑ *x_i_* is equal to the number of cases and *n* − ∑ *x_i_* is equal to the number of controls.

Henceforth, we assume that *F* is inverse-logit. To test the hypothesis of no genotype/phenotype association at a specific marker *H*_0_ : θ*_G_* = 0 (or equivalently, *H*_0_ : π_0_ = π_1_ = π) we use the score test statistic

(2) $$Z_{G}=\frac{\sum_{i=1}^{n}\left(x_{i}-\bar{x}\right)\left(g_{i}-\hat{\pi }\right)}{\sqrt{n\bar{x}(1-\bar{x})\hat{\pi }(1-\hat{\pi })}}=\frac{T_{G}}{D_{G}},$$

where $\bar{x}=\frac{1}{n}\sum x_{i}$ and $\hat{\pi }$ = *g* (introduced for example in Agresti, 2012). In a study with an equal number of cases and controls we have *x* = 1/2 and thus, the above simplifies to

(3) $$Z_{G}=\frac{1}{2}\frac{\sqrt{n}\left({\hat{\pi }}_{1}-{\hat{\pi }}_{0}\right)}{\sqrt{\hat{\pi }(1-\hat{\pi })}},$$

where ${\hat{\pi }}_{1}$ and ${\hat{\pi }}_{0}$ are the sample means for *g* in the cases and controls, respectively. Under the null hypothesis θ*_G_* = 0, the random variable *Z_G_* has mean 0 and variance 1 and its distribution is approximately normal for sufficiently large *n*.

In addition to *G*, consider a second marker *H* and let ξ_*x*_ = Pr (*H* = 1|*x*). As in Equation (2) above, the relevant score statistic is

(4) $$Z_{H}=\frac{\sum_{i=1}^{n}\left(x_{i}-\bar{x}\right)\left(h_{i}-\hat{\xi }\right)}{\sqrt{n\bar{x}(1-\bar{x})\hat{\xi }(1-\hat{\xi })}}=\frac{T_{H}}{D_{H}}$$

with $\hat{\xi }$ = *h*. Setting E(*H*) = ξ, the conditional distribution of *H* given *G* is *p*_*h*|*g*_ = Pr (*H* = *h*|*G* = *g*), providing a measure of the LD between G and H. Consequently ξ = *p*_1|0_ + π (*p*_1|1_ − *p*_1|0_) and

(5) $$\text{cor}(G,H)=\left(p_{1|1}-p_{1|0}\right){\left\{\frac{\pi (1-\pi )}{\xi (1-\xi )}\right\}}^{1/2}.$$

In the following, we derive the relation between the correlation of the test statistics *Z_G_* and *Z_H_* and the correlation between *G* and *H* under the null hypothesis (θ*_G_* = 0) and local alternatives, and defer the derivations for global alternatives to the supplementary material.

Under the null hypothesis of no association, cov(*Z_G_*, *Z_H_*) = *E* (*T_G_T_H_*). Using Equations (2) and (4) we have that

(6) $$\begin{array}{l} E\left(T_{G}T_{H}\right)=E\left\{\sum_{i}\left(x_{i}-\bar{x}\right)\left(g_{i}-\hat{\pi }\right)\times \sum_{i}\left(x_{i}-\bar{x}\right)\left(h_{i}-\hat{\xi }\right)\right\}\\ \;=E\left\{E\left[\sum_{i}\left(x_{i}-\bar{x}\right)\left(g_{i}-\hat{\pi }\right)\times \sum_{i}\left(x_{i}-\bar{x}\right)\left(h_{i}-\hat{\xi }\right)|g\right]\right\}\\ \;=E\{\sum_{i}\left(x_{i}-\bar{x}\right)\left(g_{i}-\hat{\pi }\right)\\ \;\times \sum_{i}\left(x_{i}-\bar{x}\right)\left(p_{1|g_{i}}-\left[p_{1|0}+\hat{\pi }\times \left(p_{1|1}-p_{1|0}\right)\right]\right)\} \end{array}$$

The last line follows from

(7) $$E\left\{\left(h_{i}-\hat{\xi }\right)|g_{i}\right\}=\left(1-\hat{\xi }\right)p_{1|g_{i}}-\hat{\xi }\text{​}\left(1-p_{1|g_{i}}\right)=p_{1|g_{i}}-\hat{\xi }.\;$$

Assuming that the participants are unrelated and that $\hat{\pi }$ ≡ π, the above expectation simplifies to

(8) $$\begin{array}{l} E\left(T_{G}T_{H}\right)=E\{\sum_{i}{\left(x_{i}-\bar{x}\right)}^{2}\left(g_{i}-\pi \right)\\ \;\times \left\{\left(p_{1|g_{i}}-\left[p_{1|0}+\pi \times \left(p_{1|1}-p_{1|0}\right)\right]\right)\right\}\}\\ \;=\sum_{i}{\left(x_{i}-\bar{x}\right)}^{2}E\left\{\left(g_{i}-\pi \right)p_{1|g_{i}}\right\}\\ \;=n\bar{x}(1-\bar{x})E\left(g_{i}p_{1|g_{i}}\right)-\pi E\left(p_{1|g_{i}}\right)\\ \;=n\bar{x}(1-\bar{x})\left(p_{1|1}-p_{1|0}\right)\pi (1-\pi ), \end{array}$$

which is equal to zero if *p*_1|0_ = *p*_1|1_ = *p* (no linkage between *G* and *H*), and equal to *n* *x* (1 − *x*) π (1 − π) if *p*_1|0_ = 0 and *p*_1|1_ = 1 (perfect linkage). If π and ξ were known then the denominators *D_G_* and *D_H_* are constants, and thus

(9) $$\begin{array}{l} \text{cor}(Z_{G},Z_{H})=\frac{n\bar{x}(1-\bar{x})\left(p_{1|1}-p_{1|0}\right)\pi (1-\pi )}{n\bar{x}(1-\bar{x})\sqrt{\pi (1-\pi )\xi (1-\xi )}}\\ \;=\left(p_{1|1}-p_{1|0}\right){\left\{\frac{\pi (1-\pi )}{\xi (1-\xi )}\right\}}^{1/2}\\ \;=\text{cor}(G,H). \end{array}$$

Thus, under the null hypothesis and local alternatives, subject to the approximation that $\hat{\pi }$ and $\hat{\xi }$ are constants, the correlation between the test statistics at two markers equals the correlation between the marker genotypes. If there is no correlation between *G* and *H* (linkage equilibrium) and *H* is not independently causal, then *H* is not associated with disease status. If *H* is in LD with *G*, an association with the disease status is induced by *G*.

For a local alternative ($\theta_{G}=O(1/\sqrt{n})$) a first-order Taylor series approximation yields

(10) $$\begin{array}{l} Z_{G}\approx N\text{​}\left(\Delta_{G},1\right)\text{ with}\\ \Delta_{G}=\theta_{G}{\left\{n\bar{x}(1-\bar{x})\hat{\pi }(1-\hat{\pi })\right\}}^{1/2} \end{array}$$

If *G* is “causal” for the trait of interest but *H* is not, then the correlation between *G* and *H* induces a non-zero θ*_H_* in the score test. Specifically,

(11) $$\begin{array}{l} Z_{H}\approx N\left(\Delta_{H},1\right)\text{ with}\\ \Delta_{H}=\theta_{H}{\left\{n\bar{x}(1-\bar{x})\hat{\xi }(1-\hat{\xi })\right\}}^{1/2}\\ \;=\theta_{G}\times \left(p_{1|1}-p_{1|0}\right){\left\{n\bar{x}(1-\bar{x})\hat{\xi }(1-\hat{\xi })\right\}}^{1/2}, \end{array}$$

where the last line follows from $\hat{\xi }$ = *p*_1|0_ + $\hat{\pi }$ × (*p*_1|1_ − *p*_1|0_). Note that Δ*_H_* is induced, so that in case of linkage equilibrium (*p*_1|1_ = *p*_1|0_) we have Δ*_H_* = 0. Also note that θ*_H_* depends on both the odds ratio at the causal marker (θ*_G_*) and the covariation of the genotypes.

### Multi-marker tests for association

Let **Z** = (*Z*_1_, …, *Z_K_*) be the score test Z statistics from an LD block with *K* markers.

#### The maximum z-statistic *Z*_max_

We define the maximum z-statistic as *Z*_max_ = max_1 ≤ *k* ≤ *K*_ {|*Z*_*k*_|}. The null distribution of *Z*_max_ depends on the correlation matrix **R** of the test statistics **Z**, and for large samples we have **Z** ~ *N_K_*(0, **R**). The two-sided *p*-value for *Z*_max_ can be derived from this multivariate distribution by calculating

(12) $$p_{\text{max}}=2\times \left\{1-\Phi_{R}(Z_{\text{max}}{}^{\circ}1_{K})\right\}$$

where Φ_**R**_ is the cumulative distribution function of the multivariate normal distribution with mean vector 0 and correlation matrix **R**, **1_K_** is a vector of ones of length *K*, and the symbol ° denotes the dot product.

#### The Bonferroni corrected *p*-value

We compute the Bonferroni *p*-value in a set of *K* markers as *K* times the *p*-value stemming from the most significant marker as given by *Z*_max_ = max_1≤*k*≤*K*_ {|*Z*_*k*_|}.

#### The optimal linear combination *Z*_opt_

We consider a block of correlated markers as the region of interest and assume that one of these SNPs is biologically associated with the trait of interest. The statistical associations at neighboring markers are thus controlled by the strength of the correlation between the causal marker and other loci in the analysis. With locus-specific Z-scores being approximately normally distributed, a linear combination (**L**′ **Z**) is optimal. Generalizing from the two-locus case to a block of *K* SNPs with π_*k*_ = pr(*G*_*k*_ = 1) for *k* ∈ {1, …, *K*}, we define

(13) $$\begin{array}{l} \Delta_{k}=E(Z_{k})=\theta_{k}B_{k}\\ B_{k}={\left\{n\bar{x}(1-\bar{x}){\hat{\pi }}_{k}\left(1-{\hat{\pi }}_{k}\right)\right\}}^{1/2} \end{array}$$

The values *B_k_* are known and depend on the minor allele frequency, but in general the θ_*k*_ are unknown. The optimal linear combination depends on the relative sizes of the Δ_*k*_ and so an assumption on the relative sizes of the θ_*k*_ is needed, and certain cases are discussed below and in the next section. We let **Z** denote the vector of the *Z_k_* and **Δ** denote the vector of the Δ_*k*_.

We need to identify the *K*-dimensional vector **L**_opt_ that maximizes the non-centrality {*E*(**L**′ **Z**)}^2^ = **L**′**ΔΔ**′**L** subject to **L**′**RL** = 1, with the correlation matrix **R** computed from the genotype correlation structure. This is equivalent to finding the $\tilde{L}$ that maximizes $\tilde{L}$′ **H** $\tilde{L}$ subject to $\tilde{L}$′ $\tilde{L}$ = 1, where **L** = **R**^−1/2^ $\tilde{L}$ and **H** = **R**^−1/2^**ΔΔ**′**R**^−1/2^. A standard matrix theory result (which can formally be derived using Lagrange multipliers) yields that $\tilde{L}$ is the normalized first principal component loading vector of **H**, and we have **L**_opt_ = **R**^−1/2^ $\tilde{L}$ so that *Z*_opt_ = **L**′_opt_ **Z**.

Note that **L**_opt_ depends only on the relative sizes of the **Δ**s. If Δ_*k*_ ≡ Δ, then **L**_opt_ = **R**^−1^ **1** / (**R**^−1^_++_)^**1**/**2**^ where **R**^−1^_++_ is the sum of all entries of **R**^−1^. Further, in the case that all minor allele frequencies in the block are the same, then all *B_k_* are identical and **Δ** is the product of a constant and the row (or column) of **R** corresponding to the locus which is biologically associated with the trait of interest. (This follows from an extension of Equations (10) and (11) to more than two markers.) In this case, **H** has a degenerate form with all but one entry (the entry on the diagonal position corresponding to that of the causal locus) equal to 0. This yields that **L**_opt_ is simply a vector equal to 1 at the causal locus, and zero otherwise, i.e., *Z*_opt_ = *Z*_*causal*_ for sets of markers with equal minor allele frequency. Thus, even though the associations in the remaining markers of the block are only induced by the causal SNP, there is in general more information in the optimal linear combination of score statistics than in the statistic from the causal locus alone, since the minor allele frequencies across a set of markers are virtually always non-identical, unless the markers are in perfect LD (and thus, every marker contains the same information about statistical association with the phenotype).

We also note that the expectation of the optimal linear combination is *E*(*Z*_opt_) = **L**′_opt_ **Δ** = (**Δ**′ **R**^−1^**Δ**)^1/2^, and thus the non-centrality is

(14) $${\left\{E\left(Z_{\text{opt}}\right)\right\}}^{2}={\left(L_{\text{opt}}^{\prime }\Delta \right)}^{2}=\left(\Delta^{\prime }R^{-1}\Delta \right).$$

If *K* is very large, care is needed in computing **R**^−1^. However, in most situations either **R** will be relatively small (limited to the size of an LD block) or will have considerable structure with many zeros and non-communicating subsets and so only matrices of small to medium size will need to be inverted.

#### The “agnostic” linear combination *Z*_eq_

As in the case of the optimal linear combination *Z*_opt_ above we consider a linear combination of z-scores, albeit without any prior knowledge of the location of any causal variant. This lack of knowledge comes into play in choosing a value of **Δ**, and we assume a uniform prior over the set of possible causal variants. In this case, the expected non-centrality is

$${\left\{E\left(L^{\prime }Z\right)\right\}}^{2}=L^{\prime }\left[\frac{1}{K}\sum_{k}\Delta^{\left(k\right)}{\left[\Delta^{\left(k\right)}\right]}^{\prime }\right]L$$

where Δ^(*k*)^ is the vector of expected values of the test statistics, assuming that marker *k* is the causal marker. More specifically, Δ^(*k*)^ is the *k*^th^ column of **R** that has been component-wise multiplied by the *B_k_*s. In this case, we proceed as described above to find **L**_opt_, with our matrix **H** given by

$$H=R^{-1/2}\left[\frac{1}{K}\sum_{k}\Delta^{\left(k\right)}{\left[\Delta^{\left(k\right)}\right]}^{\prime }\right]R^{-1/2}$$

This approach maximizes the pre-posterior expected non-centrality, although this does not guarantee better performance than *Z*_max_.

#### The sequence kernel association test (SKAT)

For comparison with the above methods, we also include the sequence kernel association test (SKAT), a widely used method for SNP-set analysis based on a logistic kernel-machine approach that allows for flexible, covariate adjusted relations between a genotype and the outcome of interest (Wu et al., 2010). Analyses were carried out using the publicly available SKAT R package (http://cran.r-project.org/web/packages/SKAT) with default settings that produce a linearly weighted kernel, with weights inversely proportional to minor allele frequency.

## Results

### Simulations based on assumed LD and allele frequencies

We simulated a “naive” population under a dominant disease model using the R package bindata (http://cran.r-project.org/web/packages/bindata). We simulated 5-locus haplotype blocks with exchangeable (compound symmetry, CS) and auto-regressive lag-1 (AR1) correlation structures, with correlations between 0 and 0.8. For all markers in the haplotype blocks we chose constant minor allele frequencies in this simulation, set at either 5 or 25%. One causal marker was selected and haplotypes were sampled to generate cases and controls as given by the genetic risk model, using a variety of odds ratios (1, 1.1, 1.4, and 1.7). We generated 50,000 samples of 1000 cases and 1000 controls, and carried out marker-specific score tests to generate sets of test statistics.

We investigated the type I error and power for the approach using the maximum z statistic, the optimal linear combination of the test statistics with known causal locus (comb_opt_), and the “agnostic” linear combination of the test statistics assuming equal prior probabilities for each marker in the block to be causal (comb_eq_). In addition, to mimic some limited biological information available, we show results for a linear combination of test statistics assuming equal prior probabilities for the causal and one additional marker, narrowing the set of potentially causal markers to two out of five (comb_pair_). For the compound symmetry simulations each pair of markers that contains the causal one is equivalent. For the auto-regressive lag-1 simulations, we show a pair of markers with correlation *ρ* and a pair of markers with correlation *ρ*^2^. Estimates of type I error and power are the fraction of simulations with *p*-values lower than the set significance level assuming two-sided tests. We also include the results derived for the Bonferroni correction with the significance level divided by the number of markers assessed. In addition to the typical significance level α = 0.05, we also assessed the different methods using a much stricter significance level for type I error control, as is usually done in GWAs. These extreme tail probabilities were estimated using importance sampling (see supplementary material).

**Figure 1:**
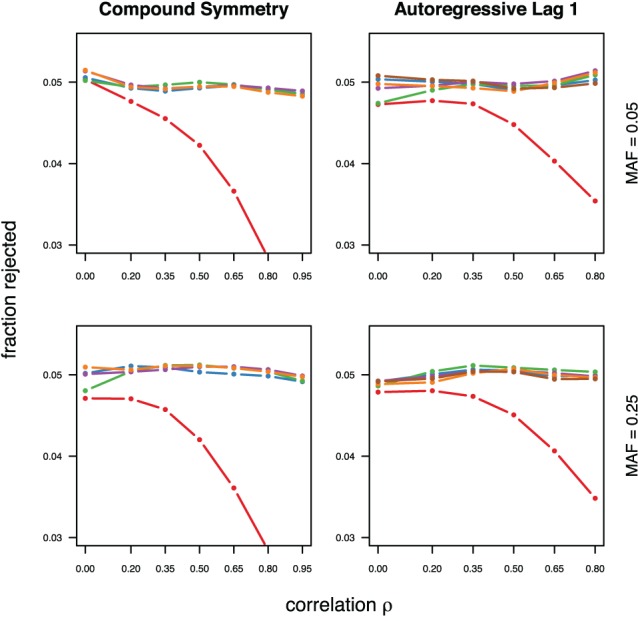
**Calibration under the null hypothesis for compound symmetry (left) and auto-regressive (right) correlation structures, assuming equal minor allele frequencies of 5% (top) and 25% (bottom) in a block of 5 markers**. The fraction of rejected null hypotheses of no association (estimated type I error, y-axis) simulated in 50,000 data sets each is shown as a function of the between-marker correlation (x-axis). Each line represents a different method (see the following figures for details), with the Bonferroni method (red) showing the sharp decline as *ρ* increases.

With the exception of the conservative Bonferroni correction all methods were well calibrated under the null hypothesis, for both types of correlations (compound symmetry and auto-regressive) and both minor allele frequencies considered (Figure 1). For much stronger type-I error control however all approaches can be slightly conservative, in particular in settings with auto-regressive correlation structure (see supplementary material).

**Figure 2:**
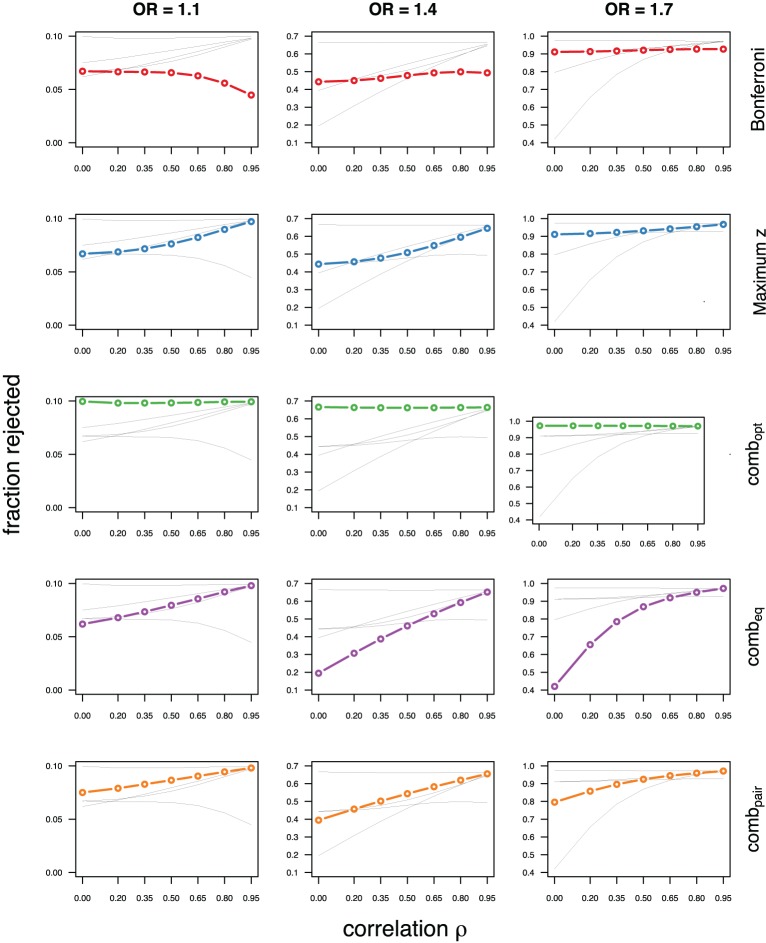
**Comparing analytic strategies in the setting with compound symmetry correlation structures, assuming equal minor allele frequencies of 5% in a block of 5 markers**. The fraction of rejected null hypotheses of no association (power, y-axis) in 50,000 simulated data sets is shown as a function of the between-marker correlation (x-axis) for assumed odds-ratios of 1.1 (**left**), 1.4 (**middle**), and 1.7 (**right**). Each row highlights a different method, as labeled along the right-hand side.

**Figure 3:**
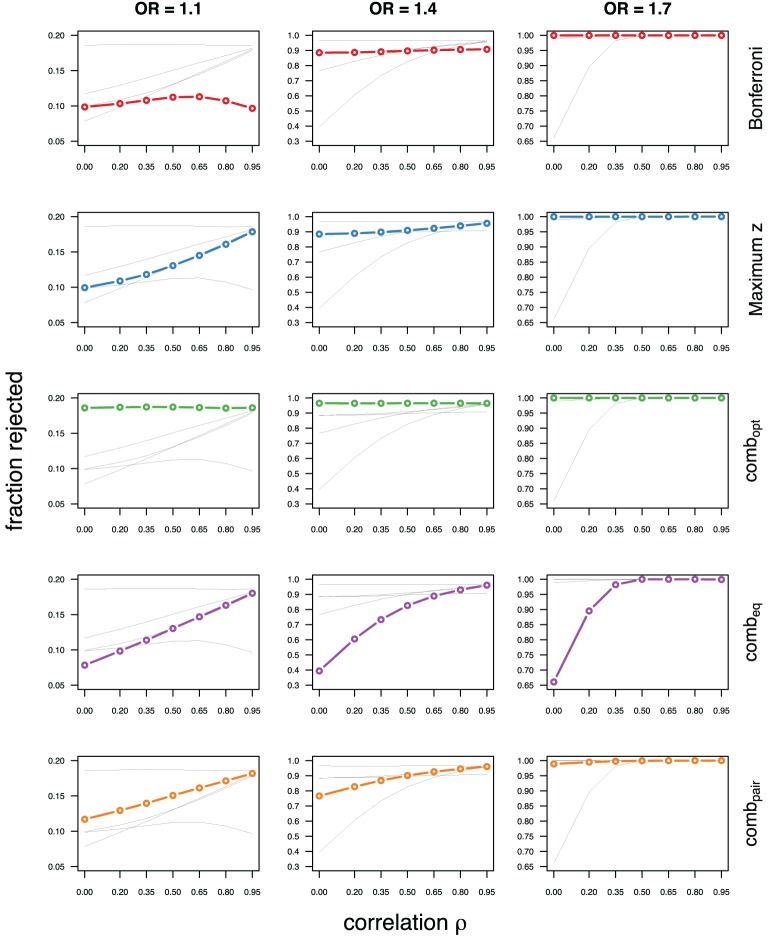
**Comparing analytic strategies in the setting with compound symmetry correlation structures, assuming equal minor allele frequencies of 25% in a block of 5 markers**. The fraction of rejected null hypotheses of no association (power, y-axis) in 50,000 simulated data sets is shown as a function of the between-marker correlation (x-axis) for assumed odds-ratios of 1.1 (**left**), 1.4 (**middle**), and 1.7 (**right**). Each row highlights a different method, as labeled along the right-hand side.

**Figure 4:**
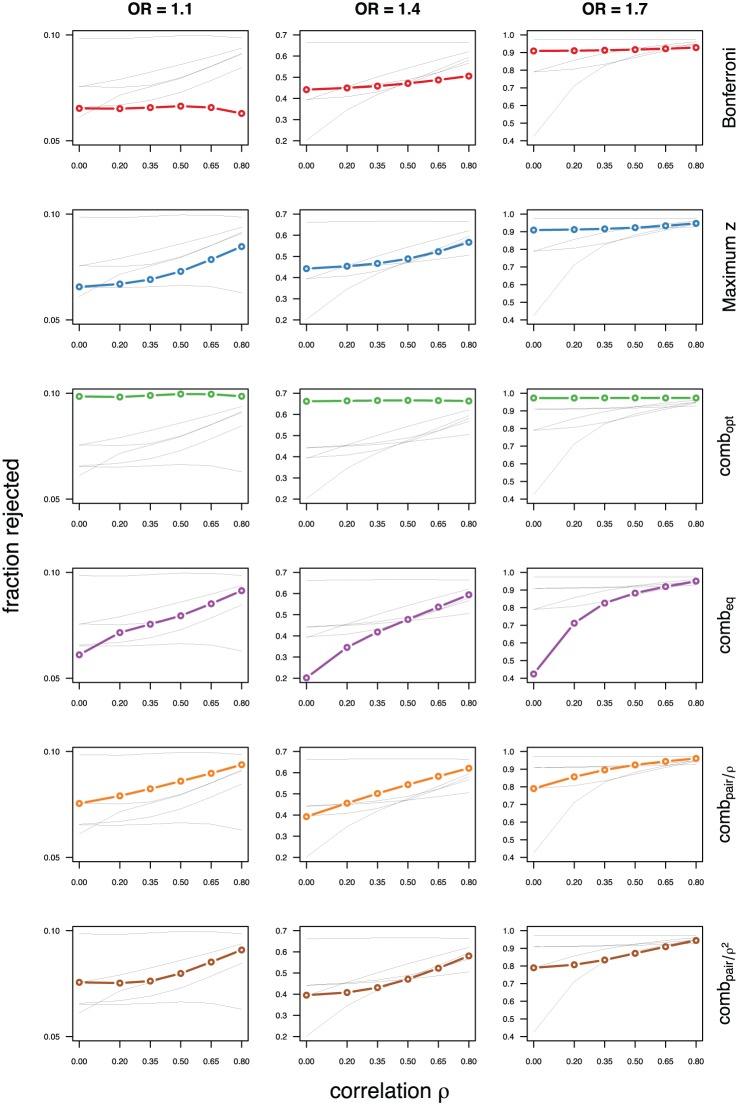
**Comparing analytic strategies in the setting with autoregressive correlation structures, assuming equal minor allele frequencies of 5% in a block of 5 markers**. The fraction of rejected null hypotheses of no association (power, y-axis) in 50,000 simulated data sets is shown as a function of the between-marker correlation (x-axis) for assumed odds-ratios of 1.1 (**left**), 1.4 (**middle**), and 1.7 (**right**). Each row highlights a different method, as labeled along the right-hand side.

**Figure 5:**
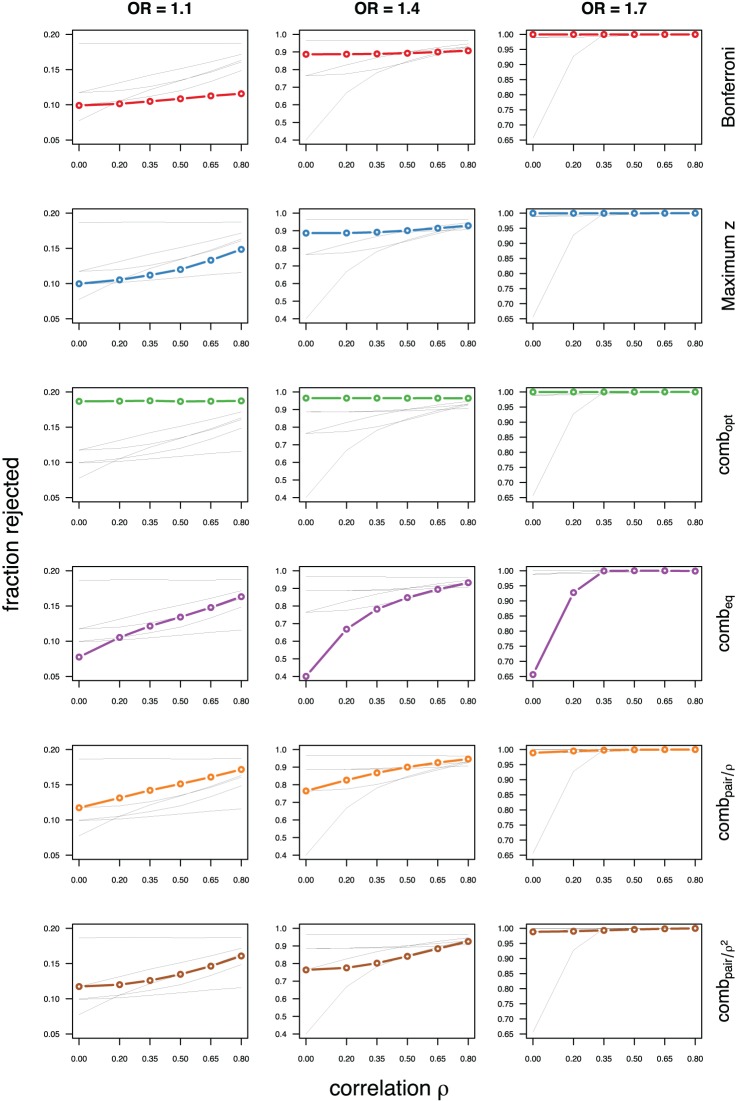
**Comparing analytic strategies in the setting with autoregressive correlation structures, assuming equal minor allele frequencies of 25% in a block of 5 markers**. The fraction of rejected null hypotheses of no association (power, y-axis) in 50,000 simulated data sets is shown as a function of the between-marker correlation (x-axis) for assumed odds-ratios of 1.1 (**left**), 1.4 (**middle**), and 1.7 (**right**). Each row highlights a different method, as labeled along the right-hand side.

As expected, the optimal linear combination with correctly specified causal locus outperforms all other methods, yielding the largest power for odds-ratios of 1.1, 1.4, and 1.7. For this method, the estimated power was virtually constant for all magnitudes of correlations across markers within a block, for both simulated compound symmetry at low (Figure 2) and high (Figure 3) minor allele frequencies, as well as auto-regressive correlation structures (Figures 4, 5, respectively). Also as expected, the relative loss of power for the other methods is worst for uncorrelated markers, and decreases with increasing correlation. For perfectly correlated markers, all methods except Bonferroni are equivalent. The data-driven maximum z-statistic which does not require any biological knowledge or other input generally performs better than comb_eq_ and Bonferroni, and thus, is a practical and more powerful method than conventionally employed approaches.

Not surprisingly, the relative power of the Bonferroni method is particularly poor for highly correlated markers with compound symmetry when the genetic signal is weak (Figures 2, 3, left column). If the prior information about the locus for the causal variant is not very strong, little if any improvement can be achieved compared to the maximum z-statistic method. In the hypothetical case when the causal locus can be narrowed down to one of two loci in the LD block, comb_pair_ occasionally yields modestly higher power than the maximum z-statistic method, but this is typically only the case when the two markers are in strong LD. However, for large effect sizes and weak correlations in the LD block, comb_pair_ performs at times even worse than multiple comparison correction via Bonferroni, and particularly noticeable at low minor allele frequencies (Figures 2, 4, right columns). As expected, comb_pair_ with *ρ* always yields higher power than comb_pair_ with *ρ*^2^in the auto-regressive setting (Figures 4, 5). The performance of comb_eq_ is arguably the worst, and particularly poor in settings with high minor allele frequencies and strong signal (Figures 3, 5, right columns).

### Simulations based on genomic data

As realistic examples of LD and minor allele frequencies, we also simulated data based on haplotypes in two regions of chromosome 10 and one region on chromosome 22 delineated from the genome scans (Illumina Human660W-Quad BeadChip array) of 4251 European American participants in the Lung Health Study, a NHLBI-supported multi-center randomized clinical trial in the United States and Canada to determine whether or not a program of smoking intervention and use of an inhaled bronchodilator could slow the rate of decline in pulmonary function in smokers with mild airflow limitation (Kanner et al., 1999).

On chromosome 10 we chose two smaller blocks of 8 and 7 markers respectively (top of Table 1 with lower minor allele frequencies and weaker LD; top of Table 2 with larger minor allele frequencies and stronger LD). On chromosome 22 we chose a larger block of 24 markers (mean *R*^2^of 0.35, minor allele frequencies between 0.07 and 0.41, median of 0.28, see supplementary material). This block is part of a previously identified region of strong LD observed in a Caucasian population (Dawson et al., 2002). We used inferred haplotypes in these regions to simulate case-control data sets with varying degrees of association between a hypothetical causal locus and the phenotype. For each block and a set of five different effect sizes (odds-ratios of 1, 1.1, 1.25, 1.4, and 1.7) we generated 50,000 samples of 1000 cases and 1000 controls, and carried out marker-specific score tests to generate sets of test statistics.

**Table 1A:** **Linkage disequilibrium between SNPs measured by Pearson's correlation coefficient along with the SNP minor allele frequencies in an eight marker LD block on chromosome 10, simulated based on genome scans of samples from the Lung Health Study**.

	**1**	**2**	**3**	**4**	**5**	**6**	**7**	**8**
1	1	0.93	0.57	0.78	0.47	0.45	0.07	0.43
2		1	0.61	0.81	0.46	0.49	0.08	0.47
3			1	0.50	−0.14	−0.13	0.29	0.29
4				1	0.68	0.67	0.14	0.53
5					1	0.96	−0.09	0.38
6						1	−0.07	0.39
7							1	0.59
8								1
MAF	0.25	0.22	0.10	0.28	0.17	0.16	0.21	0.43

**Table 1B:** **Estimated type-I errors (for *OR* = 1.00) and power (*OR* = 1.10, 1.25, 1.40 and 1.70) for different methods of addressing multiple comparisons in the eight marker LD block**.

**Odds ratio**	**1.00**	**1.10**	**1.25**	**1.40**	**1.70**
Bonferroni	0.035	0.099	0.520	0.904	1.000
Maximum Z	0.050	0.128	0.576	0.925	1.000
comb_opt_	0.051	0.184	0.688	0.958	1.000
comb_eq_	0.049	0.090	0.266	0.515	0.869
comb_pair_ (markers 5 and 4)	0.051	0.121	0.442	0.774	0.986
comb_pair_ (markers 5 and 7)	0.050	0.061	0.114	0.192	0.328
SKAT	0.048	0.058	0.118	0.282	0.799

*Marker 5 was assumed to be the causal locus*.

**Table 2A:** **Linkage disequilibrium between SNPs measured by Pearson's correlation coefficient along with the SNP minor allele frequencies in a seven marker LD block on chromosome 10, simulated based on genome scans of samples from the Lung Health Study**.

	**1**	**2**	**3**	**4**	**5**	**6**	**7**
1	1	0.99	−0.96	−0.83	−0.83	0.76	−0.69
2		1	−0.97	−0.84	−0.84	0.77	−0.69
3			1	0.81	0.81	−0.74	0.69
4				1	0.95	−0.84	0.76
5					1	−0.87	0.77
6						1	−0.67
7							1
MAF	0.49	0.49	0.49	0.44	0.45	0.49	0.44

**Table 2B:** **Estimated type-I errors (for *OR* = 1.00) and power (*OR* = 1.10, 1.25, 1.40 and 1.70) for different methods of addressing multiple comparisons in the seven marker LD block**.

**OR**	**1.00**	**1.10**	**1.25**	**1.40**	**1.70**
Bonferroni	0.023	0.184	0.843	0.997	1.000
Maximum Z	0.036	0.234	0.879	0.998	1.000
comb_opt_	0.051	0.321	0.937	1.000	1.000
comb_eq_	0.051	0.288	0.904	0.998	1.000
comb_pair_ (markers 4 and 3)	0.051	0.293	0.910	0.999	1.000
comb_pair_ (markers 4 and 6)	0.051	0.299	0.916	0.999	1.000
SKAT	0.051	0.298	0.920	0.999	1.000

*Marker 4 was assumed to be the causal locus*.

In the eight marker block with the lower minor allele frequencies and weaker LD, we observed that all methods were well-calibrated under the null hypothesis (odds ratio equal to 1), with the exception of the conservative Bonferroni correction. The optimal linear combination with correctly specified causal locus again outperformed all other methods, yielding the largest power for odds-ratios of 1.1, 1.25, 1.4, and 1.7 (bottom of Table 1). The data-driven maximum z-statistic which does not require biological knowledge or other input showed a slight loss in power compared to the optimal method, however performed substantially better than any of the other approaches considered, including the hypothetical cases where the causal locus could be narrowed down to one of two loci (comb_pair_ in Table 1), even when those two markers were in somewhat strong LD (correlation of 0.68 between marker 4 and the causal locus 5). In this example, the “paired” approach performed even worse than multiple comparison correction via Bonferroni.

For the seven marker block with the larger minor allele frequencies and stronger LD we observed similar results (bottom of Table 2). However, the hypothetical case where the causal locus could be narrowed down to one of two loci yielded higher power when the two markers were in strong LD (correlation of 0.81 between marker 3 and the causal locus 4). Interestingly, the sequence kernel association test showed very different performances for these two simulation scenarios. While properly calibrated under the null, the power for the simulation on the block of eight markers with overall lower minor allele frequencies and weaker LD was substantially lower than the power of most of the other methods (Table 1). On the other hand, for the simulation on the block of seven markers with larger minor allele frequencies and stronger LD, the performance was very competitive. One possible explanation for this behavior is the weighting scheme in SKAT - by default, low frequency variants carry higher weights than common variants. In the first setting, the marker with the lowest minor allele frequency (i.e., the highest weight) has only a very weak correlation to the causal, much more variable SNP (*ρ* = −0.14), while in the second setting all markers have about the same minor allele frequency, and are much more strongly correlated.

The simulation on the 24 marker block yields similar results in general, but some qualitative differences are noteworthy. The optimal linear combination again performs best overall, although for odds ratios of 1.4 the maximum z-statistic shows slightly higher power (Table 3), likely due to the somewhat fragmented LD across the 24 markers observed in our population (see supplementary figures). As before, the “paired” approach performs well when the two markers are in strong LD (the causal markers 12 and marker 13 have an *R*^2^of 0.97), and yields unsatisfactory power when the markers are not in strong LD (*R*^2^of 0.05 between markers 12 and 3). The performance of SKAT again is affected by the distribution of minor allele frequencies in the block and the observed LD. While the causal marker 12 has an appreciable minor allele frequency of 0.28, some other markers show much less variation, and thus receive more weight in the default settings. Here, the lowest minor allele frequency (MAF = 0.07) is observed for marker 3, which is in very low LD with the causal marker (*R*^2^of 0.05). Overall, and similar to previous results, the largest gain of power for the optimal linear combination relative to the other methods is seen at lower odds ratios.

**Table 3:** **Estimated type-I errors (for *OR* = 1.00) and power (*OR* = 1.10, 1.25, 1.40 and 1.70) for different methods of addressing multiple comparisons in a 24 marker block on chromosome 22, simulated based on genome scans of samples from the Lung Health Study (correlation and LD structure shown in the supplementary materials)**.

**OR**	**1.00**	**1.10**	**1.25**	**1.40**	**1.70**
Bonferroni	0.026	0.107	0.634	0.967	1.000
Maximum Z	0.047	0.159	0.717	0.979	1.000
comb_opt_	0.051	0.199	0.741	0.974	1.000
comb_eq_	0.050	0.137	0.528	0.860	0.996
comb_pair_ (markers 12 and 13)	0.050	0.192	0.721	0.968	1.000
comb_pair_ (markers 12 and 3)	0.048	0.072	0.187	0.361	0.689
SKAT	0.050	0.063	0.136	0.283	0.710

*Minor allele frequencies in the region ranged from 0.07 to 0.41, and marker 12 (MAF 0.28) was assumed to be the causal locus. Marker 13 has a MAF of 0.28 and R^2^ of 0.96 with marker 12, marker 3 has a MAF of 0.07 and R^2^ of 0.04 with marker 12*.

## Discussion

We evaluated three approaches to controlling multiplicity in GWAs: standard Bonferroni, the correlation-calibrated maximum statistic, and a theoretical benchmark: the optimal linear combination of locus specific test statistics which requires knowledge of the causal locus. Computation of the latter two depends on the correlation among the test statistics; the performance of each depends on this correlation. We reiterate that the correlation among the test statistics is essentially identical to the biological correlation amongst the genotypes (the LD structure) and this can be estimated. For an additional comparison to the above methods, we included the sequence kernel association test (SKAT), a widely used method for SNP-set analysis based on a logistic kernel-machine approach that allows for flexible, covariate adjusted relations between a genotype and the outcome of interest.

Simulations show that the two correlation-dependent approaches are well-calibrated under the null hypothesis. As expected, unless the correlations are very small, the Bonferroni approach is conservative. In the context of the test Z-scores being well approximated by a multivariate normal distribution, the optimal linear combination dominates all other approaches, but this optimality is quite fragile, depending on having identified the causal locus or one in high LD with it. If the causal locus is poorly selected, our linear combination using “best guess” weights as one example, the properly-calibrated Max statistic often performs better, sometimes by a substantial amount with these relations depending on the magnitude of the correlations, their pattern (compound symmetry or auto-regressive), and the magnitude of the genotype-phenotype association. We do see gains in power using a linear combination where we have narrowed down the set of candidate loci in our block, particularly in the case of very small effect sizes. The haplotype-based simulations produce similar comparisons, but with generally smaller differences amongst the approaches.

Overall, the calibrated maximum method is very effective at maintaining power compared to use of a linear combination. However, when the causal locus is correctly specified, the optimal linear combination can confer a considerable increase in power. Therefore, there is some room for error and we have also provided an approach by which it is possible to specify prior probabilities on the loci and then use the induced, optimal linear combination. As we have shown, if there are two markers in a block that have a higher prior likelihood of being associated with disease (e.g., due to damaging functional prediction), putting higher weights, or all weight, on these will provide robustness to mis-specification of the causal locus, while providing more power than the Max test in some cases, especially for very low effect sizes. However, our equally weighted case is equivalent to giving each locus equal prior probability and its generally poor performance indicates that some focus is needed.

We also found that the sequence kernel association test (SKAT) run with its default values is a very competitive method in settings when LD within a block is strong—which also implies similar minor allele frequencies between markers, as high *R*^2^values are mathematically not possible between SNPs of very different allele frequencies. On the other hand, the power in the simulations with lower minor allele frequencies and weaker LD was lower for SKAT than the power of both the maximum and the optimal linear combination tests. We conjecture that this is due to the default weighting scheme in SKAT—up-weighting less common variants—while in our simulation the marker with the lowest minor allele frequency and thus the highest weight had only a very weak correlation to the assumed causal marker. Thus, we believe that the default SKAT is most useful for blocks with high LD, and for association tests under the common assumption of higher penetrance for lower allele frequency variants. We also note that SKAT allows for weighted burden tests, which we did not consider in this manuscript.

One challenge for all methods of this type is the dependence on having pre-defined blocks of interest. There are several existing methods for estimating LD-structures (e.g., Stephens et al., 2001; Gabriel et al., 2002; Browning and Browning, 2009) which can be used to identify LD-blocks. Here, we have estimated the LD by computing correlation values of the encoded genotypes using the data set at hand, rather than external databases, which avoids incorporating mis-specified structure due to differences in sample populations compared to an external reference. This is in contrast to the often recommended usage of external data, and it will be informative to investigate in detail the impact of ambiguous LD blocks (such as the 24 marker block from chromosome 22) for any of the considered methods.

### Conflict of interest statement

The authors declare that the research was conducted in the absence of any commercial or financial relationships that could be construed as a potential conflict of interest.

## References

[B1] Agresti A. (2012). Categorical Data Analysis. New York, NY: Wiley, 3rd Edn

[B2] Ballard D. H. Cho J. Zhao H. (2010). Comparisons of multi-marker association methods to detect association between a candidate region and disease. Genet. Epidemiol. 34, 201–212 10.1002/gepi.20448 19810024 PMC3158797

[B3] Basu S. Pan W. Shen X. Oetting W. S. (2011). Multilocus association testing with penalized regression. Genet. Epidemiol. 35, 755–765 10.1002/gepi.20625 21922539 PMC3350336

[B4] Browning B. L. Browning S. R. (2009). A unified approach to genotype imputation and haplotype-phase inference for large data sets of trios and unrelated individuals. Am. J. Hum. Genet. 84, 210–223 10.1016/j.ajhg.2009.01.005 19200528 PMC2668004

[B5] Chai H.-S. Sicotte H. Bailey K. R. Turner S. T. Asmann Y. W. Kocher J.-P. A. (2009). GLOSSI: a method to assess the association of genetic loci-sets with complex diseases. BMC Bioinformat. 10:102 10.1186/1471-2105-10-102 19344520 PMC2678095

[B6] Chapman J. Whittaker J. (2008). Analysis of multiple SNPs in a candidate gene or region. Genet. Epidemiol. 32, 560–566 10.1002/gepi.20330 18428428 PMC2691454

[B7] Chapman J. M. Cooper J. D. Todd J. A. Clayton D. G. (2003). Detecting disease associations due to linkage disequilibrium using haplotype tags: a class of tests and the determinants of statistical power. Hum. Hered. 56, 18–31 10.1159/000073729 14614235

[B8] Chasman D. I. (2008). On the utility of gene set methods in genomewide association studies of quantitative traits. Genet. Epidemiol. 32, 658–668 10.1002/gepi.20334 18481796

[B9] Conneely K. N. Boehnke M. (2007). So many correlated tests, so little time! Rapid adjustment of P values for multiple correlated tests. Am. J. Hum. Genet. 81, 1158–1168 10.1086/522036 17966093 PMC2276357

[B10] Dawson E. Abecasis G. R. Bumpstead S. Chen Y. Hunt S. Beare D. M. (2002). A first-generation linkage disequilibrium map of human chromosome 22. Nature 418, 544–548 10.1038/nature00864 12110843

[B11] Fisher R. A. (1932). Statistical Methods for Research Workers. 4th Edn Edinburgh: Oliver and Boyd

[B12] Gabriel S. B. Schaffner S. F. Nguyen H. Moore J. M. Roy J. Blumenstiel B. (2002). The structure of haplotype blocks in the human genome. Science 296, 2225–2229 10.1126/science.1069424 12029063

[B13] Gauderman W. J. Murcray C. Gilliland F. Conti D. V. (2007). Testing association between disease and multiple SNPs in a candidate gene. Genet. Epidemiol. 31, 383–395 10.1002/gepi.20219 17410554

[B14] Genz A. Bretz F. (2009). Computation of Multivariate Normal and t Probabilities. Lecture Notes in Statistics. Heidelberg: Springer-Verlag 10.1007/978-3-642-01689-9

[B15] Genz A. Bretz F. Miwa T. Mi X. Leisch F. Scheipl F. (2011). mvtnorm: Multivariate Normal and t Distributions. R package version 0.9-9991.

[B16] Goeman J. J. Van De Geer S. A. Van Houwelingen H. C. (2006). Testing against a high dimensional alternative. J. R. Stat. Soc. Ser. B 68, 477–493 10.1111/j.1467-9868.2006.00551.x

[B17] Holden M. Deng S. Wojnowski L. Kulle B. (2008). GSEA-SNP: applying gene set enrichment analysis to SNP data from genome-wide association studies. Bioinformatics 24, 2784–2785 10.1093/bioinformatics/btn516 18854360

[B18] Horne B. D. Camp N. J. (2004). Principal component analysis for selection of optimal SNP-sets that capture intragenic genetic variation. Genet. Epidemiol. 26, 11–21 10.1002/gepi.10292 14691953

[B19] Kanner R. E. Connett J. E. Williams D. E. Buist A. S. (1999). Effects of randomized assignment to a smoking cessation intervention and changes in smoking habits on respiratory symptoms in smokers with early chronic obstructive pulmonary disease: the Lung Health Study. Am. J. Med. 106, 410–416 10.1016/S0002-9343(99)00056-X 10225243

[B20] Kwee L. C. Liu D. Lin X. Ghosh D. Epstein M. P. (2008). A powerful and flexible multilocus association test for quantitative traits. Am. J. Hum. Genet. 82, 386–397 10.1016/j.ajhg.2007.10.010 18252219 PMC2664991

[B21] Li J. Ji L. (2005). Adjusting multiple testing in multilocus analyses using the eigenvalues of a correlation matrix. Heredity 95, 221–227 10.1038/sj.hdy.6800717 16077740

[B22] Li M. Wang K. Grant S. F. A. Hakonarson H. Li C. (2009). ATOM: a powerful gene-based association test by combining optimally weighted markers. Bioinformatics 25, 497–503 10.1093/bioinformatics/btn641 19074959 PMC2642636

[B23] Makambi K. C. A. (2003). Weighted inverse chi-square method for correlated significance tests. J. Appl. Stat. 30, 225–234 10.1080/0266476022000023767

[B24] Moskvina V. Schmidt K. M. (2008). On multiple-testing correction in genome-wide association studies. Genet. Epidemiol. 32, 567–573 10.1002/gepi.20331 18425821

[B25] Mukhopadhyay I. Feingold E. Weeks D. E. Thalamuthu A. (2010). Association tests using kernel-based measures of multi-locus genotype similarity between individuals. Genet. Epidemiol. 34, 213–221 10.1002/gepi.20451 19697357 PMC3272581

[B26] Nyholt D. R. (2004). A simple correction for multiple testing for single-nucleotide polymorphisms in linkage disequilibrium with each other. Am. J. Hum. Genet. 74, 765–769 10.1086/383251 14997420 PMC1181954

[B27] O'Dushlaine C. Kenny E. Heron E. A. Segurado R. Gill M. Morris D. W. (2009). The SNP ratio test: pathway analysis of genome-wide association datasets. Bioinformatics 25, 2762–2763 10.1093/bioinformatics/btp448 19620097

[B28] Pan W. (2011). Relationship between genomic distance-based regression and kernel machine regression for multi-marker association testing. Genet. Epidemiol. 35, 211–216 10.1002/gepi.20567 21308765 PMC3134543

[B29] Pan W. Han F. Shen X. (2010). Test selection with application to detecting disease association with multiple SNPs. Hum. Hered. 69, 120–130 10.1159/000264449 19996609 PMC3725887

[B30] Schaid D. J. McDonnell S. K. Hebbring S. J. Cunningham J. M. Thibodeau S. N. (2005). Nonparametric tests of association of multiple genes with human disease. Am. J. Hum. Genet. 76, 780–793 10.1086/429838 15786018 PMC1199368

[B31] Schaid D. J. Rowland C. M. Tines D. E. Jacobson R. M. Poland G. A. (2002). Score tests for association between traits and haplotypes when linkage phase is ambiguous. Am. J. Hum. Genet. 70, 425–434 10.1086/338688 11791212 PMC384917

[B32] Seaman S. R. Müller-Myhsok B. (2005). Rapid simulation of p values for product methods and multiple-testing adjustment in association studies. Am. J. Hum. Genet. 76, 399–408 10.1086/428140 15645388 PMC1196392

[B33] Shi W. Lee K. E. Wahba G. (2007). Detecting disease-causing genes by LASSO-Patternsearch algorithm. BMC Proc. 1(Suppl. 1):S60 Available online at: http://www.biomedcentral.com/1753-6561/1/S1/S60 18466561 10.1186/1753-6561-1-s1-s60 PMC2367607

[B34] Stephens M. Smith N. J. Donnelly P. (2001). A new statistical method for haplotype reconstruction from population data. Am. J. Hum. Genet. 68, 978–989 10.1086/319501 11254454 PMC1275651

[B35] Wang K. Li M. Bucan M. (2007). Pathway-based approaches for analysis of genomewide association studies. Am. J. Hum. Genet. 81, 1278–1283 10.1086/522374 17966091 PMC2276352

[B36] Wang T. Elston R. C. (2007). Improved power by use of a weighted score test for linkage disequilibrium mapping. Am. J. Hum. Genet. 80, 353–360 10.1086/511312 17236140 PMC1785334

[B37] Wang T. Jacob H. Ghosh S. Wang X. Zeng Z.-B. (2009a). A joint association test for multiple SNPs in genetic case-control studies. Genet. Epidemiol. 33, 151–163 10.1002/gepi.20368 18770519 PMC2719721

[B38] Wang X. Zhang S. Sha Q. (2009b). A new association test to test multiple-marker association. Genet. Epidemiol. 33, 164–171 10.1002/gepi.20369 18720476 PMC3572742

[B39] Wu M. C. Kraft P. Epstein M. P. Taylor D. M. Chanock S. J. Hunter D. J. (2010). Powerful SNP-set analysis for case-control genome-wide association studies. Am. J. Hum. Genet. 86, 929–942 10.1016/j.ajhg.2010.05.002 20560208 PMC3032061

[B40] Wu T. T. Chen Y. F. Hastie T. Sobel E. Lange K. (2009). Genome-wide association analysis by lasso penalized logistic regression. Bioinformatics 25, 714–721 10.1093/bioinformatics/btp041 19176549 PMC2732298

[B41] Xiong M. Zhao J. Boerwinkle E. (2002). Generalized T2 test for genome association studies. Am. J. Hum. Genet. 70, 1257–1268 10.1086/340392 11923914 PMC447600

