# Supplementary material for: On multi-marker tests for association in case-control studies
Source: Front Genet. 2013 Dec 16;4:252. doi: 10.3389/fgene.2013.00252 (PMC3863805; doi:10.3389/fgene.2013.00252)
Supplement: Supplementary file 1 [file Presentation1.PDF]

On multi-marker tests for association in case-control studies

Supplementary Material

Margaret A. Taub[1], Holger R. Schwender[2], Samuel G. Younkin[1],
Thomas A. Louis[1], Ingo Ruczinski[1]

[1]Department of Biostatistics, Johns Hopkins University, Baltimore, Maryland, USA.
[2]Mathematical Institute, Heinrich Heine University Düsseldorf, Germany.

Web Appendix A: More General Methods

Global correlation structure

We study the marginal distribution of Z_G and Z_H and their association by evaluating the mean, variance and covariance of T_G and T_H. All computations are conditional on the binary disease indicator $\mathbf{x}$. For convenience, we define $\pi(\bar{x}) = \bar{x}\pi_1 + (1-\bar{x})\pi_0$ and $\xi(\bar{x}) = \bar{x}\xi_1 + (1-\bar{x})\xi_0$.

$$\begin{aligned} E(T_G) &= \sum_i (x_i - \bar{x})\{g_i - \pi(\bar{x})\} = n\bar{x}(1-\bar{x})(\pi_1 - \pi_0) \\ &= 0, \text{ if } \pi_0 = \pi_1 = \pi = \pi(\bar{x}) \qquad (1) \\ V(T_G) &= \sum_i (x_i - \bar{x})^2 \{\pi_0(1-\pi_0)(1-x_i) + \pi_1(1-\pi_1)x_i\} \\ &= n\bar{x}(1-\bar{x})\{\bar{x}\pi_0(1-\pi_0) + (1-\bar{x})\pi_1(1-\pi_1)\} \\ &= n\bar{x}(1-\bar{x})\pi(1-\pi) = D_G^2, \text{ if } \pi_0 = \pi_1 = \pi \end{aligned}$$

Note that $\bar{x}$ multiplies the π_0 term. Also, the ratio of $V(T_G)$ to D_G^2, provides the non-null $V(Z_G)$.

Similarly,

$$\begin{aligned} E(T_H) &= n\bar{x}(1-\bar{x})(\xi_1 - \xi_0) = n\bar{x}(1-\bar{x})(\pi_1 - \pi_0)(p_{1|1} - p_{1|0}) \\ &= 0, \text{ if either } \pi_0 = \pi_1 \text{ or } p_{1|0} = p_{1|1} \text{ (no LD)} \qquad (2) \\ V(T_H) &= n\bar{x}(1-\bar{x})\{\bar{x}\xi_0(1-\xi_0) + (1-\bar{x})\xi_1(1-\xi_1)\} \\ &= n\bar{x}(1-\bar{x})\xi(1-\xi) = D_H^2, \text{ if either } \pi_0 = \pi_1 \text{ or } p_{1|0} = p_{1|1} \text{ (no LD)} \end{aligned}$$

Computations similar to the foregoing give:

$$\begin{aligned} \text{cov}(T_G, T_H) &= \sum_i (x_i - \bar{x})^2 \{x_i\pi_1(1-\pi_1)(p_{1|1} - p_{1|0}) + (1-x_i)\pi_0(1-\pi_0)(p_{1|1} - p_{1|0})\} \qquad (3) \\ &= n\bar{x}(1-\bar{x})(p_{1|1} - p_{1|0})\{(1-\bar{x})\pi_1(1-\pi_1) + \bar{x}\pi_0(1-\pi_0)\} \end{aligned}$$

Combining (3) with (1) and (2), we obtain:

$$\text{cor}(T_G, T_H) = (p_{1|1} - p_{1|0}) \left(\frac{\bar{x}\pi_0(1-\pi_0) + (1-\bar{x})\pi_1(1-\pi_1)}{\bar{x}\xi_0(1-\xi_0) + (1-\bar{x})\xi_1(1-\xi_1)} \right)^{\frac{1}{2}}$$

The correlation of G and H is:

$$\text{cor}(G, H) = (p_{1|1} - p_{1|0}) \left(\frac{\pi(\bar{x})(1-\pi(\bar{x}))}{\xi(\bar{x})(1-\xi(\bar{x})} \right)^{\frac{1}{2}}.$$

As in the local case, if $\pi_0 = \pi_1$ (the null holds), then $\text{cor}(T_G, T_H) = \text{cor}(G, H)$. More generally,

$$\left[\frac{\text{cor}(T_G, T_H)}{\text{cor}(G, H)} \right]^2 = \frac{\left(\frac{\bar{x}\pi_0(1-\pi_0)+(1-\bar{x})\pi_1(1-\pi_1)}{\pi(\bar{x})(1-\pi(\bar{x}))} \right)}{\left(\frac{\bar{x}\xi_0(1-\xi_0)+(1-\bar{x})\xi_1(1-\xi_1)}{\xi(\bar{x})(1-\xi(\bar{x})} \right)}.$$

Trend-test formulation for general genotype encoding

The derivation in the main text assumes binary genotype encoding (dominant/recessive), however here we consider a more general trend-test model with $G \in \{0, 1, 2\}$, and $H \in \{0, 1, 2\}$, and the correlation of the two Z scores as induced by the association of G and H.

In this setting, we define $d_\nu =$ to be the score used in the trend test for $G = \nu$ or $H = \nu$ (without loss of generality (wlog) $d_0 = 0$). We could set $d_1 = 1$, also wlog, so long as it is to be given a non-zero score, because the trend test is invariant to location/scale changes in the scores. However, retaining a general d_1 makes it easier to follow the math and allows for complete flexibility for the genomic model. Similarly, we define r_ν to be the score given to $G = \nu$ or $H = \nu$, when computing the correlation between G and H (wlog, $r_0 = 0$, because correlations are location/scale invariant). As for the d's, we could set $r_1 = 1$, but retain the general notation.

We define $\pi_{g|x} = \text{pr}(G = g \mid x)$, $\pi_{+|x} = 1$, and similarly, $\xi_{h|x} = \text{pr}(H = h \mid x)$, as the conditional probabilities of genotype given phenotype. We also define the unconditional probabilities $\pi_g = \text{pr}(G = g)$ and $\pi_h = \text{pr}(H = h)$. To quantify the correlation between G and H, we define $p_{h|g} = \text{pr}(H = h \mid G = g)$.

We want to test the hypothesis H_0: $\pi_{g|0} = \pi_{g|1} = \pi_g, g \in \{0, 1, 2\}$ of no genotype/phenotype association

at the causal SNP and use a score test statistic as above:

$$\begin{aligned} Z_G &= \frac{\sum_i (x_i - \bar{x})[d_1\{\mathbf{1}(g_i = 1) - \pi_1\} + d_2\{\mathbf{1}(g_i = 2) - \pi_2\}]}{[n\bar{x}(1-\bar{x})\{d_1^2\pi_1(1-\pi_1) - 2d_1d_2\pi_1\pi_2 + d_2^2\pi_2(1-\pi_2)\}]^{\frac{1}{2}}} \\ &= T_G/D_G \end{aligned}$$

where $\mathbf{1}()$ is the indicator function. Similarly, for the other SNP we obtain,

$$\begin{aligned} Z_H &= \frac{\sum_i (x_i - \bar{x})[d_1\{\mathbf{1}(h_i = 1) - \xi_1\} + d_2\{\mathbf{1}(h_i = 2) - \xi_2\}]}{[n\bar{x}(1-\bar{x})\{d_1^2\xi_1(1-\xi_1) - 2d_1d_2\xi_1\xi_2 + d_2^2\xi_2(1-\xi_2)\}]^{\frac{1}{2}}} \\ &= T_H/D_H \end{aligned}$$

Expected values and variances of T_G and T_H can be calculated in a straightforward manner, for example

$$\begin{aligned} E(T_G) &= n\bar{x}(1-\bar{x})[d_1(\pi_{1|1} - \pi_{1|0}) + d_2(\pi_{2|1} - \pi_{2|0})] \\ &= 0, \text{ if } \pi_{\nu|0} = \pi_{\nu|1} \end{aligned}$$

and

$$\begin{aligned} V(T_G) &= \sum_i (x_i - \bar{x})^2 x_i [d_1^2\pi_{1|1}(1-\pi_{1|1}) - 2d_1d_2\pi_{1|1}\pi_{2|1} + d_2^2\pi_{2|1}(1-\pi_{2|1})] \\ &\quad + \sum_i (x_i - \bar{x})^2 (1-x_i)[d_1^2\pi_{1|0}(1-\pi_{1|0}) - 2d_1d_2\pi_{1|0}\pi_{2|0} + d_2^2\pi_{2|0}(1-\pi_{2|0})] \\ &= n\bar{x}(1-\bar{x})^2[d_1^2\pi_{1|1}(1-\pi_{1|1}) - 2d_1d_2\pi_{1|1}\pi_{2|1} + d_2^2\pi_{2|1}(1-\pi_{2|1})] \\ &\quad + n\bar{x}^2(1-\bar{x})[d_1^2\pi_{1|0}(1-\pi_{1|0}) - 2d_1d_2\pi_{1|0}\pi_{2|0} + d_2^2\pi_{2|0}(1-\pi_{2|0})] \\ &= D_G^2, \text{ if } \pi_{\nu|0} = \pi_{\nu|1} \end{aligned}$$

with similar derivations for $E(T_H)$ and $V(T_H)$. Under $H_0 : \pi_{\nu|0} = \pi_{\nu|1}$ and $\xi_{\nu|0} = \xi_{\nu|1}$, so

$$\begin{aligned} \text{cov}(T_G, T_H) &= n\bar{x}(1-\bar{x}) \times \\ &\quad [d_1^2(p_{1|1} - \xi_1)\pi_1 + d_1d_2\{(p_{1|2} - \xi_1)\pi_2 + (p_{2|1} - \xi_2)\pi_1\} + d_2^2(p_{2|2} - \xi_2)\pi_2] \end{aligned}$$

which provides the numerator for $\text{cor}(T_G, T_H) = \frac{\text{cov}(T_G,T_H)}{D_G D_H}$.

The correlation of G and H is developed as follows:

$$\begin{aligned} V(G) &= r_1^2\pi_1(1-\pi_1) + r_2^2\pi_2(1-\pi_2) - 2r_1r_2\pi_1\pi_2 \\ V(H) &= r_1^2\xi_1(1-\xi_1) + r_2^2\xi_2(1-\xi_2) - 2r_1r_2\xi_1\xi_2 \\ \text{cov}(G,H) &= [\pi_1r_1(r_1p_{1|1} + r_2p_{2|1}) + \pi_2r_2(r_1p_{1|2} + r_2p_{2|2})] - \\ &\quad -\{r_1\pi_1 + r_2\pi_2\}\{r_1\xi_1 + r_2\xi_2\} \end{aligned}$$

So,

$$\text{cor}(G,H) = \frac{\text{cov}(G,H)}{\sqrt{V(G)V(H)}}$$

As in the dominant/recessive case, we have that $\text{cor}(T_G, T_H) = \text{cor}(G,H)$ under the null and local alternatives.

Importance sampling

We simulated B data sets under a model with causative odds-ratio θ_G. For each simulated data set b, we calculated the likelihood of the data under a model with parameter θ^*, denoted by $L_b(\theta^*)$, and under the null scenario, denoted by $L_b(0)$. The importance-sampling estimate of the type-1 error for a testing method T at level α is given by

$$\frac{1}{B}\sum_{b=1}^{B} \frac{\mathbf{1}\left(\text{test } T \text{ rejects at level } \alpha \text{ on data set } b\right) L_b(0)/L_b(\theta^*)}{\sum_{b=1}^{B} L_b(0)/L_b(\theta^*)},$$

where $\mathbf{1}\,()$ is the indicator function, as before.

Web Appendix B: Supplemental Figures

Illustration of induced correlation properties

In order to investigate how the relation between marker and test-statistic correlation varies under deviations from the null, we computed the ratio

$$\left[\frac{\text{cor}(T_G, T_H)}{\text{cor}(G, H)}\right]^2$$

for a variety of values of LD parameters $p_{1|0}$ and $p_{1|1}$, odds ratios θ_G, and minor allele frequencies. The ratios are generally very close to 1.0, particularly in the case of high, between-marker correlation values. The ratios deviate from 1.0 noticeably when the genotype/phenotype association is far from the null, however for the cases considered here, the ratio never drops below 0.98 (Web Figure 1).

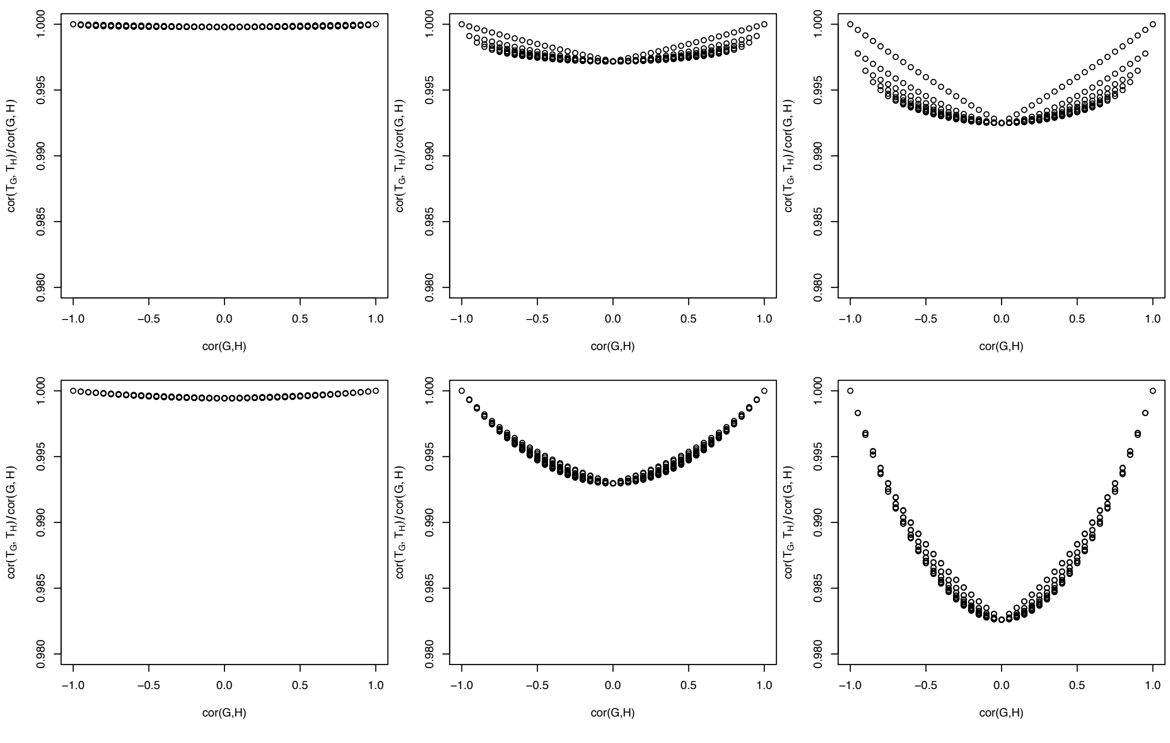

Supplementary Figure 1: Test statistic correlation ratio versus marker correlation ($p_{1|0}$ and $p_{1|1}$). Columns correspond to different levels of disease association (from left to right OR = 1.1, 1.4, and 1.7) and rows correspond to different values of MAF (top 0.05, bottom 0.25).

Linkage disequilibrium for the 24 selected markers on chromosome 22

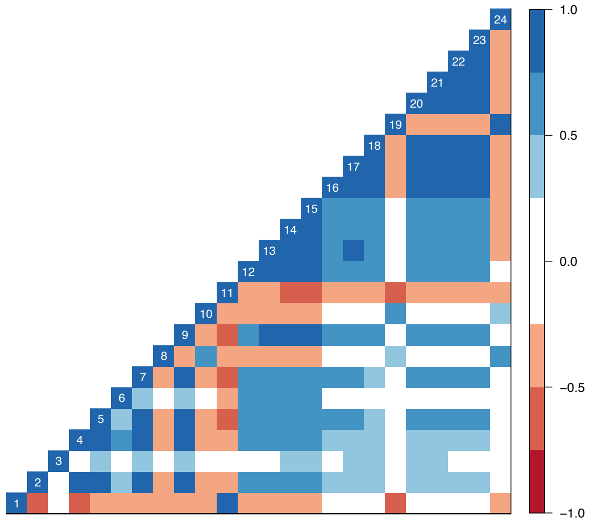

Supplementary Figure 2: Pearson's correlation for the 24 SNPs on chromosome 22 selected for the power simulation (ρ, left), and the corresponding measure of linkage disequilibrium (R^2, right).

Calibration under genome-wide significance levels

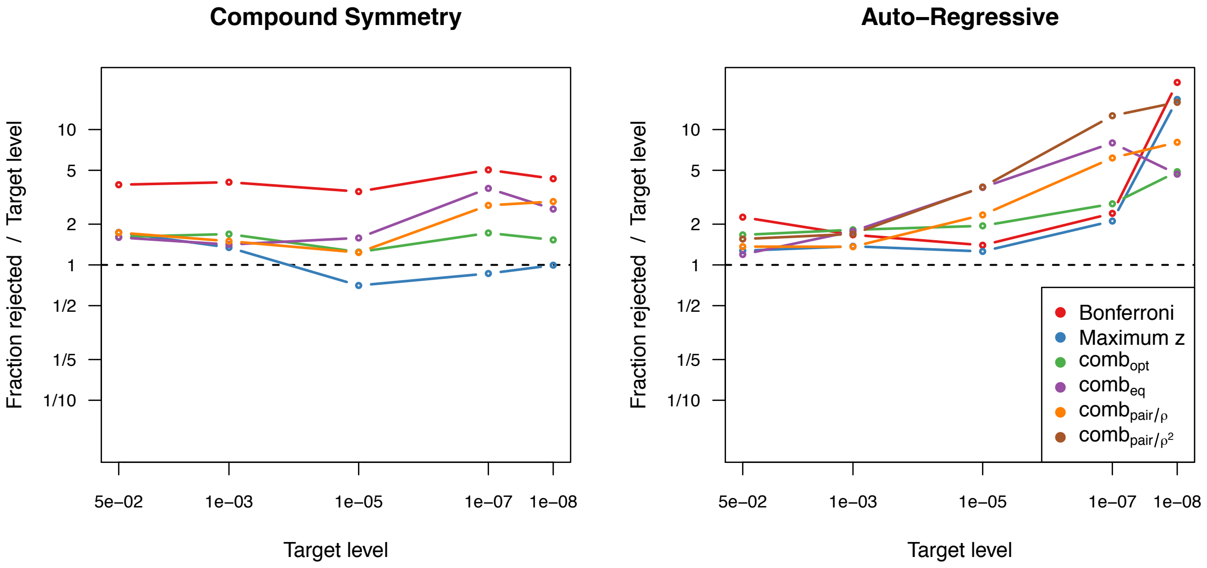

Supplementary Figure 3: Calibration under the null for compound symmetry (left) and auto-regressive (right) models, at various significance levels (x-axis).

Power under genome-wide significance levels

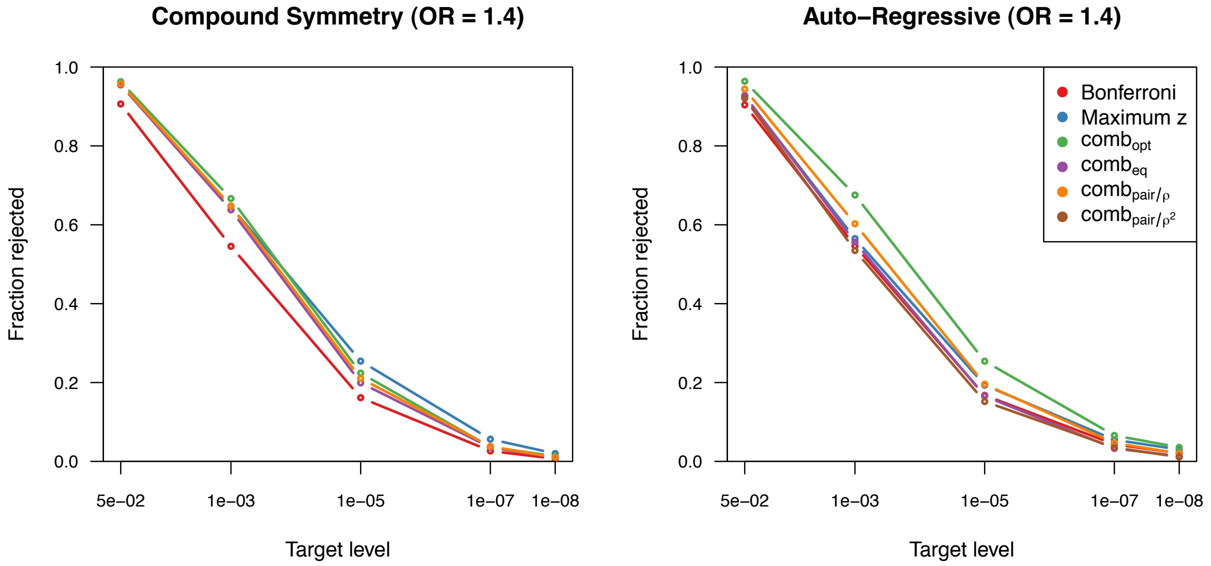

Supplementary Figure 4: Power to detect an association with odds ratios 1.4, for compound symmetry (left) and auto-regressive (right) models, at various significance levels (x-axis).

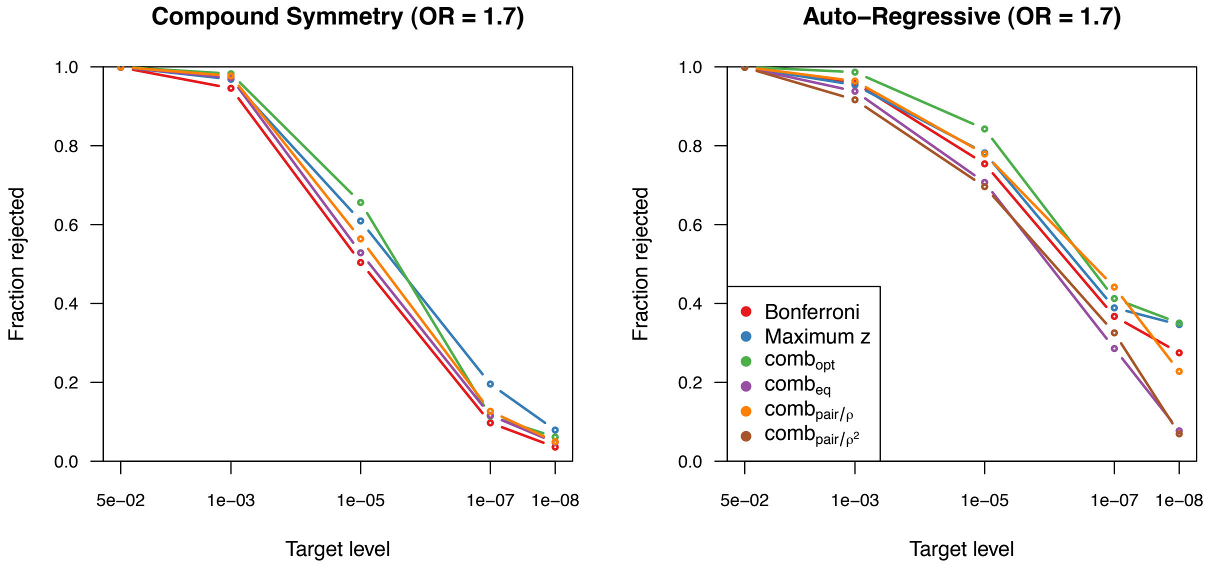

Supplementary Figure 5: Power to detect an association with odds ratios 1.7, for compound symmetry (left) and auto-regressive (right) models, at various significance levels (x-axis).
